# Multi-Center Agent Loss for Visual Identification of Chinese Simmental in the Wild

**DOI:** 10.3390/ani12040459

**Published:** 2022-02-13

**Authors:** Jianmin Zhao, Qiusheng Lian, Neal N. Xiong

**Affiliations:** 1 Institute of Information Science and Technology, Yanshan University, Qinhuangdao 066004, China; lianqs@ysu.edu.cn; 2 School of Information Engineering, Inner Mongolia University of Science & Technology, Baotou 014010, China; 3 Hebei Key Laboratory of Information Transmission and Signal Processing, Yanshan University, Qinhuangdao 066004, China; 4 Department of C.S., Colorado Technical University, Colorado Springs, CO 80907, USA; xiongnaixue@gmail.com

**Keywords:** cattle identification, deep convolutional neural networks (DCNNs), deep metric learning (DML), open-set recognition, precision livestock farming

## Abstract

**Simple Summary:**

Visual identification of cattle in a realistic farming environment is helpful for real-time cattle monitoring. Based on continuous cattle detection, identification, and behavior recognition, it is possible to utilize cameras on farms within company or government networks to provide the services of production supervision, early disease detection, and animal science research for precision livestock farming. However, cattle identification in the wild is still a difficult problem due to the high similarities of different identities and the variances of the same identity as posture or perspective changes. Our proposed method based on deep convolutional neural networks and deep metric learning provides a promising approach for cattle identification and paves the way toward continuous monitoring of cattle in a nearly natural state.

**Abstract:**

Visual identification of cattle in the wild provides an essential way for real-time cattle monitoring applicable to precision livestock farming. Chinese Simmental exhibit a yellow or brown coat with individually characteristic white stripes or spots, which makes a biometric identifier for identification possible. This work employed the observable biometric characteristics to perform cattle identification with an image from any viewpoint. We propose multi-center agent loss to jointly supervise the learning of DCNNs by SoftMax with multiple centers and the agent triplet. We reformulated SoftMax with multiple centers to reduce intra-class variance by offering more centers for feature clustering. Then, we utilized the agent triplet, which consisted of the features and the agents, to enforce separation among different classes. As there are no datasets for the identification of cattle with multi-view images, we created CNSID100, consisting of 11,635 images from 100 Chinese Simmental identities. Our proposed loss was comprehensively compared with several well-known losses on CNSID100 and OpenCows2020 and analyzed in an engineering application in the farming environment. It was encouraging to find that our approach outperformed the state-of-the-art models on the datasets above. The engineering application demonstrated that our pipeline with detection and recognition is promising for continuous cattle identification in real livestock farming scenarios.

## 1. Introduction

Chinese Simmental, native to Switzerland, are the cattle mainly farmed in China due to their comprehensive performance in milk and meat production [1]. Continuous visual cattle identification in real farming environments provides an essential stage for registration, identification, and verification for real-time cattle monitoring applicable to precision livestock farming and animal science research, such as automated production monitoring, behavioral and physiological observation, health and welfare supervision, and more [2,3]. Owing to its significance, cattle identification is becoming an emerging research field of computer vision in agriculture. On account of its uniqueness, immutability, and low costs, the visual biometric identification methodology has been a promising research trend in intelligent perception for precision farming [2]. Observable biometric identifiers for cattle, including the muzzle [4,5,6], iris image [7,8], retina vascular patterns [9], and coat pattens [10,11,12,13,14,15], promote cattle identification technology from the semi-automated to automated stage. There are mainly two drawbacks recently in using biometric characteristics. The difficulties of obtaining the cattle muzzle, iris, or retina make it hard to achieve automated and continuous identification. Moreover, the inability to see the activities from a fixed view affects a series of applications that require images of multiple viewpoints, such as behavior monitoring, physiological analysis, and so on. Chinese Simmental exhibit a yellow or brown coat with intrinsic white stripes or spots on the head, body, limbs, and tails, which is visually akin to those generated from Turing’s reaction–diffusion systems. This coat pattern makes it possible to identify cattle individuals from any viewpoint. Compared with the current use of biometric features, this work utilized the coat patten of Chinese Simmental as a biometric identifier in order to perform automated visual cattle identification with an image from any viewpoint, paving the way toward continuous monitoring of cattle in a nearly natural state.

**Figure 1:**
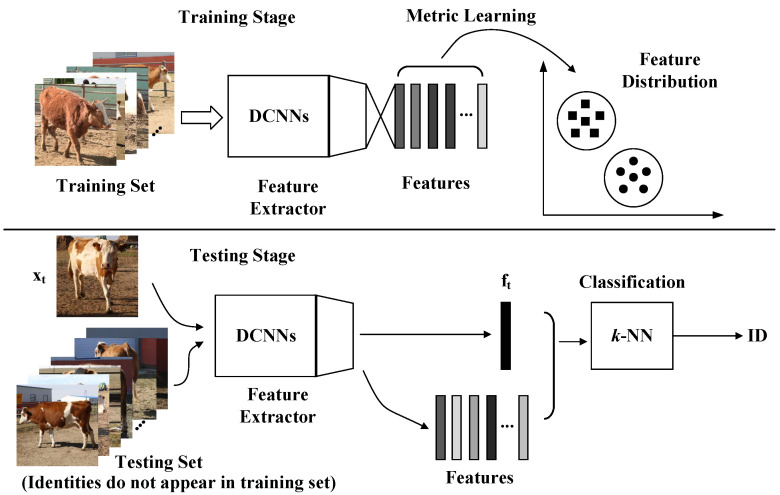
Pipeline of the cattle identification model training and testing under the open-set identification protocol. In the training stage, the deep metric learning methodology is utilized to supervise the learning process to extract separable and discriminative features. In the testing stage, the feature is extracted using DCNNs and classified by the *k*-NN classifier.

In recent years, Deep Convolutional Neural Networks (DCNNs) have achieved great success in the face recognition field and have surpassed human’s abilities on several benchmarks due to progressive network architectures and discriminative learning methods. Deep Metric Learning (DML) aims to learn the semantic embeddings by Deep Convolutional Neural Networks (DCNNs), where similar instances are closer than different ones on a manifold, and has boosted face recognition performance to an unprecedented level. In the field of visual cattle identification, DML has also been promoted and has achieved state-of-the-art performances [12,13]. The most common pipeline for visual cattle identification or face recognition under an open-set protocol involves feature extraction and classification. As shown in Figure 1, in the feature extraction stage, it is crucial to design efficient loss functions that strengthen the learning ability to obtain discriminative features in training and make it possible to obtain high performance even when the test individuals are not seen in the training stage. After training, the *k*-NN with normalized embeddings is the most commonly used classifier for identification in the testing stage.

**Figure 2:**
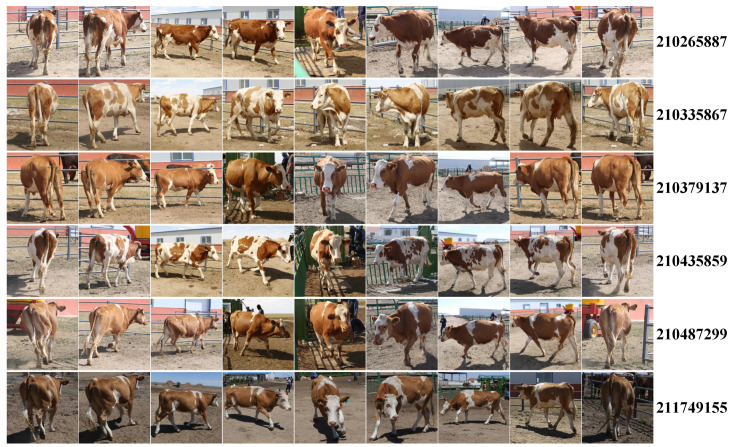
CNSID100 dataset examples. The CNSID100 dataset contains images of Chinese Simmental from multiple views, such as front, back, left, and right perspectives, on standing postures in the real farming environment for cattle identification. It contains 11,635 images of 100 identities, about 100 images with at least 3 main views per identity. Samples of several individuals in the CNSID100 dataset are given, and the most notable is the variance of imagery perspective, standing postures, and illumination conditions. The numbers on the right are the ID codes of the cattle. Best viewed in color.

The early forms of DML focused on optimizing pairwise [16] or triplet constraints [17,18,19]. Triplet loss [19], the typical DML approach, has led to state-of-the-art face recognition results by directly adding a margin among embeddings from different identities. However, there exists an obvious problem that the number of all possible pairs and triplets goes up to O($n^{2}$) and O($n^{3}$), where *n* is the number of training samples. Both contrastive and triplet constraints empirically encounter sampling difficulties in selecting informative pairs or triplets efficiently, and thus, it is difficult to learn global optimal embeddings even with a hard or semi-hard negative mining strategy. Proxy-NCA in [20] learned proxy points to construct triplets in a latent space, and it was proposed to optimize the loss with a small number of triplets, which consisted of an anchor and the similar and dissimilar proxies. However, it is very complex and inconvenient to learn proxies for triplets in the new space. Once the feature and weight vectors in the last fully connected layer are normalized to lie on a hypersphere in the SoftMax loss, the weight vector acts as a center for the features of the same class. By observing it, normalized-SoftMax-based constraints utilize this property to increase the cosine similarity among the embeddings of the same class and enforce separation among the embeddings of different classes by adding/multiplying a margin in SoftMax. A series of normalized SoftMax losses, including NormFace [21], CosFace [22], SphereFace [23], ArcFace [24], NPT loss [25], etc., has been proposed and continuously promoted performance in face recognition. In the form of normalized SoftMax loss, there is only one single center for a class; however, one naturally standing individual of Chinese Simmental has several feature clusters with the change of perspective or posture, as shown in Figure 2. Thus, a single center suffers from a lack of representing ability to obtain the diversity of information in real-world data. Softtriple loss in [26] sets multiple centers for each class to capture local clusters and has achieved State-Of-The-Art (SOTA) performance on fine-grained benchmarks. Besides clustering feature points of the same class, it is crucial to obtain sufficient separation embeddings of different individuals that are unseen in the training set for open-set identification tasks. Thus, separated centers for each class are especially essential for proxy-based constraints.

In this paper, we propose multi-center agent loss, including SoftMax with multiple centers and the *K*-nearest negative agent triplet (*K*-NNAT), by which we jointly supervise the training stage. SoftMax with multiple centers aims at reducing intra-class distance by more local centers for the embeddings to cluster. Moreover, *K*-NNAT, consisting of an embedding and its positive and *K*-nearest negative agents, directly enforces separation among different classes’ agents.

Our multi-center agent loss achieved state-of-the-art performance on the Chinese Simmental Identification dataset (CNSID100) and OpenCow2020 dataset [13] without extra mining stages. Furthermore, the engineering application significance of the proposed approach is discussed on a real-world livestock farming environment and provides a foundation for our next application research. More specifically, the contributions of this work are as follows:

(1) SoftMax loss with multi-center agents is introduced to learn the agent point for each individual to capture more local clusters of the data, and more centers for each class are helpful to reduce the intra-class variance;

(2) Multi-center agent loss consists of SoftMax with multiple centers, and *K*-NNAT loss is proposed to jointly supervise the model to learn more intra-class centers for feature clustering and to simultaneously guarantee a separation among the agents of different classes;

(3) Due to the lack of suitable datasets for the identification of cattle in a nearly natural state, the CNSID100 dataset with multi-view images was created to facilitate the experiments. It will be made available publicly after the paper is accepted to support more applications of cattle identification/re-identification and verification tasks in precision livestock farming.

The rest of the paper is organized as follows: The CNSID100 dataset is introduced in Section 3; multi-center agent loss is proposed in Section 4; the experiment details and results are provided in Section 5; finally, the conclusions and future work are given in Section 7.

## 2. Related Work

### 2.1. Visual Biometrics for Cattle Identification

Visual biometrics assign a unique identity to individual cattle according to some observable physiological characteristics [2]. With the development of computer vision technology, the identification of cattle based on visual biometric features has been one of the current and future research frontiers of computer vision in agriculture [6]. Recently, muzzle [4,5,6], iris [7,8], retina vascular pattern [9], and coat patten, including Holstein Friesian dorsal [10,11,12,13], tailhead [14], and profile images [15], have been used to perform visual cattle identification.

Cattle muzzle, a unique and permanent trait of individual cattle, has been studied as a biometric identifier for decades, from artificial features [4,5] to, recently, deep learning embeddings [6], and has pushed forward the cattle identification methodology. However, it is obvious that a muzzle image, as well as the iris and retinal vascular pattern, suffer from image capturing difficulty, especially for auto identification applications.

Comparing the above modalities, the coat pattern can be more easily obtained and thus has been utilized as a visible biometric characteristic for cattle identification recently. In [15], profile images from one side of a cow have been applied for visual identification, but single-view images are extremely limited with respect to the practicality of continuous cattle monitoring. W. Li et al. introduced the low-order Zernike moment features of cow tailhead images from a top-view camera with quadratic discriminant analysis and utilized SVM algorithms to classify the cows [14]. William Andrew et al. proposed a series of studies focusing on extracting features from full dorsal images of Holstein Friesian cattle captured by a UAV or a top-down camera, as in [14]. The works go through from exploiting manually delineated features to extracting deep learning features. More recently, Andrew et al. proposed the use of SoftMax-based reciprocal triplet loss to supervise the DCNN model learning stage and achieved promising performance for cattle identification [13]. This gives a typical standard DML approach for cattle identification under the open-set protocol.

However, to sum up the recently used visual biometric identifiers, the inconvenience of capturing muzzle, retinal, or retina vascular pattern images and the incomplete perspective of coat images from a fixed view indeed affect the continuous and automated applications such as behavior monitoring, so identification from any viewpoint of individual cattle in a natural state is needed.

### 2.2. Deep Metric Learning

Deep Metric Learning (DML) aims at mapping the raw data into the feature space such that the distance among embeddings of the same class is less than that of dissimilar identities with well-designed DCNN models and an appropriate loss function. The key ingredient is the design of efficient loss functions to learn better semantic embedding structures that keep the compactness of the same class features and guarantee separation among dissimilar individuals.

Contrastive [16] and triplet [19] constraints are typical approaches to directly obtain embeddings meeting the needs of DML, and triplet loss has become the most commonly used approach for face recognition, cattle identification, and other open-set recognition tasks. However, the extremely large set of the possible combinations of samples makes it hard to mine informative pairs or triplets to train efficiently.

In order to reduce the number of possible triplets, Proxy-NCA, as an early form of proxy triplet loss, was proposed to learn the proxies in the latent space to approximate the origin data points and construct triplets with an anchor and its positive and negative proxies in [20]. Once the feature and weight in the last fully connected layer are normalized, the SoftMax loss is used to maximize the cosine similarity between the feature and the weight. Therefore, the normalized weight vectors can be used as a representation of the class centers, and a series of normalized-SoftMax-based losses, other forms of the proxy methodology, utilize this property to achieve promising performances in face recognition tasks, including NormFace [21], SphereFace [23], CosFace [22], and ArcFace [24]. By the most extensive evaluation on over 10 face recognition benchmarks, additive angular margin loss (ArcFace), adding an angular margin penalty to the angle between the feature and its corresponding weight vector to calculate the logits in SoftMax, has become the current benchmark method in face recognition tasks [24]. Nearest-neighbors Proxy Triplet loss (NPT loss) in [25] explicitly creates a margin between an anchor and its nearest-neighbor negative weight vectors and ensures separation among different classes.

However, in all the above proxy approaches for face recognition tasks, there is only a single center for each class, not satisfying the real-world data that have multiple local clusters, especially as the samples in our CNSID100 dataset. Softtriple loss introduced multiple centers for each class to obtain the hidden clustered information of the data, and by reducing the intra-class variance, it obtained SOTA performance in fine-grained dataset benchmarks. Inspired by Softtriple and NPT loss, this work proposes multi-center agent loss, a joint supervision, to simultaneously reduce the intra-class variance by capturing inner clustered information with multiple centers for each class and keep an explicit separation among centers of different classes without any extra sampling stage.

## 3. Materials: CNSID100 Dataset

With the development of cattle identification using visual biometric identifiers, visual cattle identification or validation datasets, including muzzle, iris, and coat pattern images, have been produced. The list of datasets is shown in Table 1.

As is shown in Table 1, there is no applicable dataset for the identification of cattle with multi-view images. To facilitate the experiments carried out in this paper, we created the CNSID100 dataset, the first with multi-view images of Chinese Simmental in a natural standing state, which is much closer to the real farming environment. There are in total 11,635 images, from a population of 100 individuals, an average of above 100 images with at least 3 views per class, including front, back, left, or right. The images in the CNSID100 dataset were identified manually, and indeed, it was a time-consuming work. However, we can perform this work independently without the help of the livestock managers due to the cattle’s coat pattern. Images from some of the individuals are shown in Figure 2.

**Figure 3:**
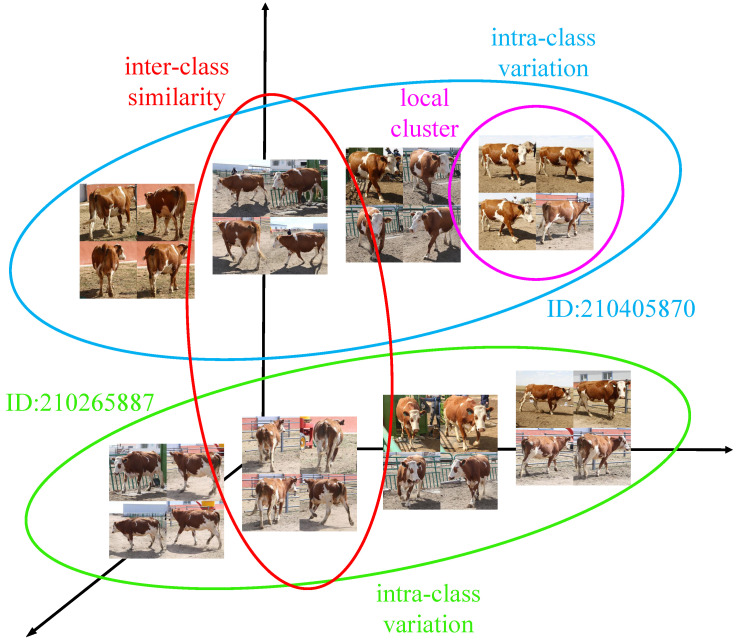
Challenges for cattle identification on the CNSID100 dataset. Samples of two typical individuals in the CNSID100 dataset are given. It is observable that the large intra-class distance, inter-class similarity, and the existence of multiple local clusters present challenges for unique identity feature extraction for Chinese Simmental identification. Best viewed in color.

As is shown in Figure 3, our dataset is much closer to the natural environment and demonstrates two main challenges in cattle identification: (1) Compared with other cattle identification or face recognition datasets, the CNSID100 dataset demonstrates a large intra-class variance with the change of views, standing postures, and illumination conditions, but very high similarity from the same-perspective images of different individuals. (2) There are several local characteristic clusters in the samples of the same identity with changes of views and standing postures, being another challenge for feature extraction in the identification task.

## 4. Methods: Our Proposed Multi-Center Agent Loss

**Figure 4:**
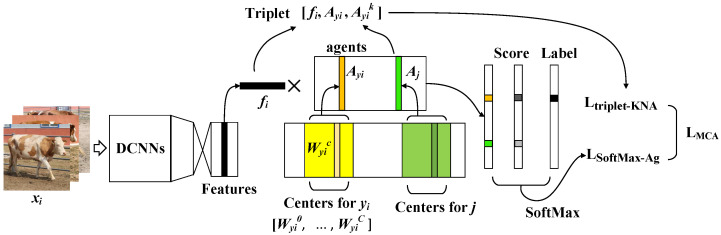
Procedures of multi-center agent loss. Multi-center agent loss includes SoftMax with multiple centers and *K*-NNAT loss. SoftMax with multiple centers uses the logit score of the agent to calculate the cross-entropy loss and learns the agent for each class. *K*-NNAT consists of the feature and its corresponding/positive agent and the *K*-nearest negative agents as hard negatives. Then, the model is jointly supervised by multi-center agent loss to learn more center points for features to concentrate on and meanwhile enforces separation among agents of different classes. Best viewed in color.

In real-world data, especially in our CNSID100 dataset, it is obvious that the large intra-class distance and small inter-class distance, as well as the existence of multiple intra-class local clusters present challenges for unique feature extraction for the identification task. Therefore, we propose multi-center agent loss to learn separable discriminative features by jointly supervising using multi-center SoftMax and agent triplet loss. The details are shown in Figure 4.

### 4.1. SoftMax with the Multi-Center Agent

It is assumed that each class has *C* centers, and as is in [26], the similarity between the feature $f_{i}$ of sample *i* and the agent of multiple centers for class $y_{i}$ is defined as,

(1) $$s_{i,y_{i}}=\max_{c=1,\cdots ,C}f_{i}^{T}W_{y_{i}}^{c},$$

where $W_{y_{i}}^{c}$ is the *c*-th weight of the multi-center [$W_{y_{i}}^{0}$,...,$W_{y_{i}}^{C}$] for class $y_{i}$.

The maximized problem in Equation (1) is considered as:

(2) $$\max_{p\in P}\sum_{c}p_{c}f_{i}^{T}W_{y_{i}}^{c}+\gamma R\left(p\right),$$

where $p\in R^{C}$ is a distribution over the class and P is a set as $P=\left(\{,p|\sum_{c}p_{c}=1,\forall c,p_{c}\geq 0,\}\right)$. R(p) is the entropy regularization of distribution *p*.

According to the K.K.T. condition [27] and the analysis in [26], *p* in Equation (2) has the closed form as:

(3) $$p_{c}=\frac{\exp(\frac{1}{\tau }f_{i}^{T}W_{y_{i}}^{c})}{\sum_{C}\exp(\frac{1}{\gamma }f_{i}^{T}W_{y_{i}}^{c})}.$$

Then, $A_{y_{i}}$, the agent of multiple centers for class $y_{i}$, is defined as:

(4) $$A_{y_{i}}=\sum_{C}p_{c}W_{y_{i}}^{c}.$$

This means that given a feature $f_{i}$ of class $y_{i}$, agent $A_{y_{i}}$ provides several local centers for $f_{i}$ to concentrate, rather than only one center for clustering. Thus, it is very helpful to reduce the intra-class variance.

In order to decrease the number of centers per class while keeping their diversity, we introduced the regularization from [26] to obtain a more sparse center matrix.

For each center $W_{i}^{t}$ for class *j*, we can make a similarity matrix as:

(5) $$S_{j}^{t}={\left[\left[W_{j}^{1}-W_{j}^{t},\cdots ,W_{j}^{K}-W_{j}^{t}\right]\right]}^{T}$$

We used the Euclidean distance to measure the similarity of two centers as ${\left(∥,W_{j}^{s}-W_{j}^{t},∥\right)}_{2}$ and the $L_{1}$-norm for $S_{j}^{t}$ to obtain a sparser center matrix for the efficient representation of local clusters. By adding the $L_{2,1}$-norm, the regularization of the multiple centers of class *j* is as:

(6) $$R\left(W_{j}^{1},\cdots ,W_{j}^{C}\right)=\sum_{t}^{C}{\left(∥,S_{j}^{t},∥\right)}_{2,1}$$

By applying the multi-center agent and regularization, SoftMax with the multi-center agent is defined as:

(7) $$\ell_{SoftMax_{Ag}}=-\log\frac{\exp(\lambda {\hat{f}}_{i}^{T}A_{y_{i}})}{\sum_{y_{i}\in Y}\exp(\lambda {\hat{f}}_{i}^{T}A_{y_{i}})}+\tau \frac{\sum_{j}^{Y}R(W_{j}^{1},\cdots ,W_{j}^{C})}{YC(C-1)},$$

where *Y* is the number of classes, *C* is the number of centers per class, τ is the scale of regularization, and λ ($\sqrt{\lambda }$ exactly) represents the radius of the hypersphere that the feature and weights are normalized to due to the problem introduced in NormFace [21], that is the existence of large gradients to the well-classified examples caused by normalization to the hypersphere with radius 1. It is noted that in the following, we use *f* to denote the normalized feature for simplicity.

The normalized SoftMax loss maximizes the cosine similarity between the feature point and its corresponding weight vector in the last fully connected layer. As was analyzed in [26], the target of our normalized SoftMax with multiple centers is:

(8) $$\forall i,j,f_{i}^{T}A_{y_{i}}\geq f_{i}^{T}A_{y_{j}}.$$

Although the SoftMax loss is designed for classification, after normalization, it is available for distance learning to constrain the cosine similarity between the feature and the positive and negative multi-center agents.

### 4.2. *K*-NNAT Loss

With the formulation of multi-center agent for each class, we introduce the triplet loss with *K*-nearest-neighbor negative agents firstly.

**Definition** **1.** *Let $A=\left\{A_{1},\cdots ,A_{Y}\right\}$, be the set of agents for Y identities. Let $f_{i}$ be an anchor feature of sample i. $A_{NN}^{\left(i\right)}=\left\{A_{1}^{\left(i\right)},A_{2}^{\left(i\right)},\cdots ,A_{K}^{\left(i\right)}\right\}$ is defined as the K-nearest negative agent set of sample i belonging to class $y_{i}$ and satisfying $d\left(f_{i},A_{1}^{\left(i\right)}\right)\leq d\left(f_{i},A_{2}^{\left(i\right)}\right)\leq \cdots \leq d\left(f_{i},A_{K}^{\left(i\right)}\right)$, for $K\neq y_{i},A_{NN}^{\left(i\right)}\subset A$.*

Then, the triplet with multi-center agent loss is given as:

(9) $$\sum_{A_{k}\in A_{NN}^{\left(i\right)}}\max\left\{0,d\left(f_{i},A_{y_{i}}\right)-d\left(f_{i},A_{k}\right)+\Delta \right\}$$

where Δ is the margin of the distance between an anchor and its positive agent and that between the anchor and any of its top-*K*-nearest negative agent. In Equation (9), only the top-*K*-nearest negative agents are used to perform the negative mining strategy without any other extra sampling manipulation.

If $f_{i}$ and $A_{k}$ are normalized to 1, we obtain:

(10) $$d\left(f_{i},A_{y_{i}}\right)-d\left(f_{i},A_{j}\right)={∥f_{i}-A_{y_{i}}∥}_{2}^{2}-{∥f_{i}-A_{j}∥}_{2}^{2}=2\left(f_{i}^{T}A_{j}-f_{i}^{T}A_{y_{i}}\right)$$

then we can reformulate our agents’ triplet loss with the cosine similarity, shown as:

(11) $$\ell_{Triplet-KNA}=\sum_{A_{k}\in A_{NN}^{\left(i\right)}}\max\left\{0,f_{i}^{T}A_{k}-f_{i}^{T}A_{y_{i}}+\Delta \right\}$$

### 4.3. Multi-Center Agent Loss

Based on the above analysis, we propose the multi-center agent loss as:

(12) $$\ell_{MCA}=\ell_{SoftMax_{Ag}}+\beta *\ell_{Triplet-KNA},$$

where β controls the learning rate of the triplet with multi-center agents. In our loss function, SoftMax with multiple centers supervises the model to learn the embedding clustering around the agent point of the corresponding class. The agent triplet loss plays the role of an implicit hard negative mining strategy because the hard negative samples are compacted to the agents with the constraints of the SoftMax part.

The properties of our proposed loss are as follows:

**Theorem** **1.** *If $\ell_{MCA}<\delta$ for $f_{i}$, then $f_{i}^{T}A_{y_{i}}-f_{i}^{T}A_{j}>\Delta -\delta$ for all j=1,2,⋯,Y, $j\neq y_{i}$.*

**Proof of Theorem 1.**  If $\ell_{MCA}<\delta$, then explicitly, $\ell_{Triplet-KNA}$. Based on the definition of $A_{NN}^{\left(i\right)}=\left\{A_{1}^{\left(i\right)},A_{2}^{\left(i\right)},\cdots ,A_{K}^{\left(i\right)}\right\}$, it is explicit that $f_{i}^{T}A_{y_{i}}-f_{i}^{T}A_{j}>f_{i}^{T}A_{y_{i}}-f_{i}^{T}A_{1}>\cdots >f_{i}^{T}A_{y_{i}}-f_{i}^{T}A_{K}>\Delta -\delta$. □

**Theorem** **2.** *If $\ell_{MCA}<\delta$ for $f_{i}$, then $d\left(A_{j},A_{y_{i}}\right)\geq 2\left(\Delta -\delta \right)$ for all j=1,2,⋯,Y, $j\neq y_{i}$.*

**Proof of Theorem 2.**  If $\ell_{MCA}<\delta$, then according to Equation (10), it has $d\left(f_{i},A_{j}\right)-d\left(f_{i},A_{y_{i}}\right)$≥2(Δ−δ). Thus, we can easily obtain $d\left(A_{j},A_{y_{i}}\right)\geq d\left(f_{i},A_{j}\right)-d\left(f_{i},A_{y_{i}}\right)\geq 2\left(\Delta -\delta \right)$ based on the triangle principle. □

Properties 1 and 2 show that our proposed loss not only guarantees the separation among the feature points and the negative agents, but also enforces a larger distance between the positive agent and its negative ones.

**Theorem** **3.** *Given $f_{i1}$, $f_{i2}$ from sample i with the same nearest negative agent $A_{k}^{\left(y_{i}\right)}$ and $f_{j}$ from sample j, with the results in* ***Property 1*** *that $f_{i}^{T}A_{y_{i}}-f_{i}^{T}A_{j}>\Delta -\delta$, if ∀i, $∥x_{i}-A_{y_{i}}∥\leq \theta$, then we have:*

(13) $${f_{i1}}^{T}f_{i2}-{f_{i1}}^{T}f_{j}=\geq \Delta -\delta -2\theta$$

**Proof of Theorem 3.** 
(14) $$\begin{array}{c} \geq {f_{i1}}^{T}\left(f_{i2}-A_{y_{i}}\right)+{f_{i1}}^{T}\left(A_{k}^{\left(i\right)}-f_{j}\right)+\delta \\ \geq \delta -{∥f_{i1}∥}_{2}{∥f_{i2}-A_{y_{i}}∥}_{2}-{∥f_{i1}∥}_{2}{∥A_{k}^{\left(i\right)}-f_{j}∥}_{2}\\ =\delta -{∥f_{i2}-A_{y_{i}}∥}_{2}-{∥A_{k}^{\left(i\right)}-f_{j}∥}_{2}\geq \Delta -\delta -2\theta \end{array}$$

□

Property 3 shows that optimizing our proposed loss with an agent margin can retain a separation among feature points of different classes. Moreover, multiple centers are very useful to obtain more local clusters for each class and reduce the intra-class distance θ.

## 5. Results

To show the performance of our proposed multi-center agent loss, a series of experiments was conducted to compare with the triplet loss [18], ArcFace [24], and Softtriple loss [26] on our CNSID100 database and SoftMax-based reciprocal triplet loss on the OpenCow2020 dataset [13]. Besides, the pipeline of cattle identification in engineering applications was verified in a real farming scenario to show the scalability of our approach to new populations and new farm scenarios. The details of the experimental analysis are presented in this section.

### 5.1. Implementation Details

The DCNN backbone architecture used in our work was ResNet50 [28], with weights pretrained on ImageNet [29]. We replaced the last fully connected layer with the inner product layer, and the output number was 384 as the dimension of the features. The images were resized to 224 × 224, and random erasing was used as the data argumentation. In the training stage, the output of the inner product layer was the features used to calculate the loss. In the testing, the input image was put into the model to obtain the features, then the features were input into the *k*-NN classifier to predict the identity.

We used Pytorch 1.7.1 to implement the multi-center agent loss. In each experiment, the network was trained on the training set of the CNSID100 dataset over 200 epochs. We used Adam as the optimizer and set the initial learning rate value as 1 × 10${}^{-3}$ for the weight updating. An exponential scheduler with γ=0.95 for the learning rate decay was utilized. The recorded accuracy was the highest value in testing after 50 epochs. Once an image was input into the network, we obtained its *d*-dimensional feature vector $f\in R^{d}$ where d=384. Then, we normalized it and used the *k*-NN algorithm with k=5 to classify the feature. To validate the model’s capability under the open-set protocol recognition tasks, we performed two-fold cross-validation on the CNSID100 dataset, with 50% individuals for training and the other half for testing. In the training stage, the unseen set was withheld, and the model only learned from the seen set. For the *k*-NN classifier, images of each identity were randomly split into training and testing samples in a ratio of 7:3. All the images were input into the network and mapped into deep features in the latent space. Then, the features of the test samples were classified with *k*-NN from the votes of the *k*-nearest features from the training samples.

### 5.2. Study of the Number of Centers

The number of centers for each class was important in our proposed multi-center agent loss function, which affected the learning efficiency and the ability to capture the variance. It is intuitive that more centers were able to obtain many local clusters that were beneficial to reducing the intra-class distance; however, too many of them dramatically increased the parameters and also reduced the representation performance of the unique centers due to their redundancy.

**Figure 5:**
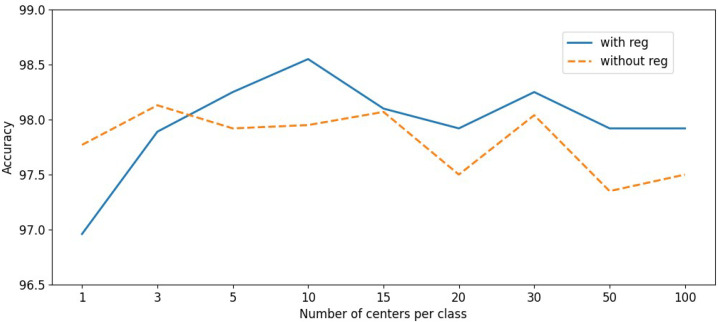
Study of the number of centers and the $L_{2,1}$ regularization. When the number of centers for each class increases to 5, the $L_{2,1}$ regularization starts to work and the accuracy decreases slightly while continuing to increase the centers to more than 10 due to the low efficiency of the redundant centers. Best viewed in color.

In Figure 5, it is shown that when the number of centers increased from C=1 to C=10, the accuracy went up to the highest value, but after that, the accuracy fell slightly while continuing to increase the centers. When the number of centers was less than five, the centers’ $L_{2,1}$-norm regularization had little effect due to the small number; however, with the increasing number of centers, its effect became more obvious.

### 5.3. Ablation Study

In order to probe the necessity of multiple centers and the agent triplet, we conducted ablation studies to demonstrate the performance of multiple centers in SoftMax and *K*-nearest negative agent triplet loss. We conducted two-fold cross-validation experiments supervised by SoftMax with a single center and a multi-center agent and equipped with the *K*-nearest negative agent triplet, respectively, to demonstrate the effectiveness of our loss with 50% individuals unseen in the CNSID100 dataset.

From Table 2, firstly, it is notable that using multiple centers was rather superior to SoftMax with a single center due to its ability to capture more local cluster information to reduce the intra-class difference. Taking multiple centers in SoftMax with or without the *K*-nearest negative agents, we could obtain an increase in the identification accuracy. Moreover, the triplet using the *K*-nearest negative agents was also helpful in SoftMax with single or multiple centers to empirically confirm the properties of our proposed loss to reduce the intra-class variance and keep the separation of different classes.

### 5.4. Comparing the Experiments

Recently, ArcFace [24] has become a benchmark in large-scale face recognition tasks. Softtriple [26] is the representation of multiple centers approach for fine-grained recognition tasks. In this section, we conducted several experiments to compare the triplet loss [18], ArcFace [24], and Softtriple loss [26] with our proposed loss. Two-fold cross-validation was employed to demonstrate the performances. For the hyperparameter selection, firstly, we chose β for the contribution of the triplet with multi-center agents. Then, we fixed β and selected γ and τ for SoftMax with multi-center loss experimentally. Finally, we conducted experiments for margin *m* in the triplet with multi-center agents for the best performance. However, λ in SoftMax with multiple centers had little effect on the performance with values of 8, 16, 24, and 32.

Consequently, for our proposed loss, we set λ=24, τ=0.2 for SoftMax with multiple centers. For multi-center agent loss, we set γ=0.1 for entropy regularization, and the center number C=10. We set a margin δ=0.4 for the top-two nearest negative agents and β=0.1. In the triplet loss, we set the margin as 0.5 and utilized the hard negative mining strategy. There are two hyperparameters in ArcFace [24]. Parameter *m* denotes the angular margin on the hypersphere and *r* is the radius of the hypersphere to which the features are normalized. With m=0.1 and r=32, we obtained the highest accuracy in the 50% identities unseen set. For Softtriple loss, we set λ=24, τ=0.2 for the $L_{2,1}$-norm, and γ=0.05 for entropy regularization, and the center number C=10. We set the margin as 0.01, the same as in [26].

As is shown in Table 3, our multi-center agent loss achieved the best performance on the CNSID100 dataset with 50% identities unseen, demonstrating the powerful supervision for intra-class local variance and inter-class separation information learning.

In [13], the OpenCow2020 dataset included indoor and outdoor cattle whole dorsal images from top-down view, made to facilitate the cattle identification experiments. We used its identification part, which consisted of 46 cows and a total of 4736 dorsal images from bird’s-eye view cameras indoors and a UAV outdoors, to compare our proposed loss with the SoftMax-based reciprocal triplet loss in [13]. SoftMax-based reciprocal triplet loss achieved the highest accuracy on the OpenCow2020 identification set with 50% individuals unseen in [13]; thus, we conducted two-fold cross-validation experiments that trained ResNet50 [28] supervised by our proposed loss.

We set C=5 and set β=0.2 and δ=0.2 to strengthen the agent triplet supervision. As can be seen in Table 4, we found that supervision with our loss function led to a margin with the same dimension of the embeddings (d=128) as in [13].

## 6. Engineering Applications

**Figure 6:**
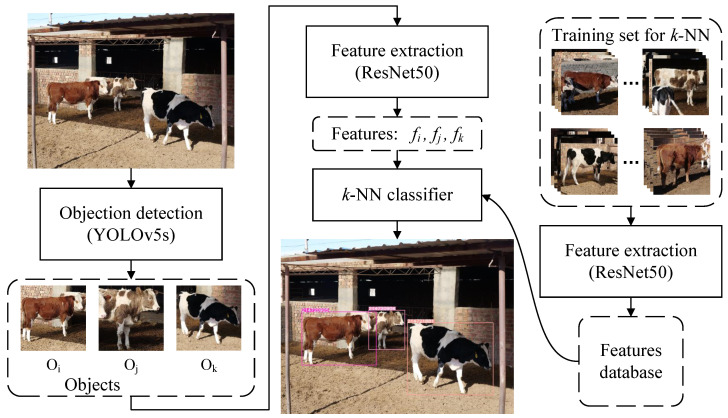
Engineering application pipeline. The images in the CNSID100 dataset were acquired partly by digital cameras, partly by smart phones, and partly by surveillance cameras at more than 12 mega pixels. Then, they were cropped with the cattle object in the center and resized to 500 × 500 pixels. In the engineering application, the image taken from the surveillance camera in the farm was input into YOLOv5s [30] to detect cattle targets. Then, the target regions were cropped, resized, and input into ResNet50 [28] to extract features for each instance. *k*-NN was used to classify the features for identification. Best viewed in color.

In order to verify the effectiveness of our proposed method in real farming scenarios, an application pipeline, including object detection, feature extraction, and identity recognition, is given in this paper. The architecture of the pipeline is shown in Figure 6. YOLOv5s [30] with the proposed weights was directly used to detect Chinese Simmental objects in the image. ResNet50 using weights trained on half of the identities in CNSID100 with the best accuracy of 98.97% in Section 5.4 was utilized as the feature extractor without retraining to show the scalability for new breeds and a real farming environment. The image taken by a real farm surveillance camera was put into the object detector to obtain the cattle targets. Then, the target regions were cropped and resized to 224 × 224 and input into the feature extractor to obtain the embeddings. The *k*-NN classifier was finally used to identify the target identity.

**Figure 7:**
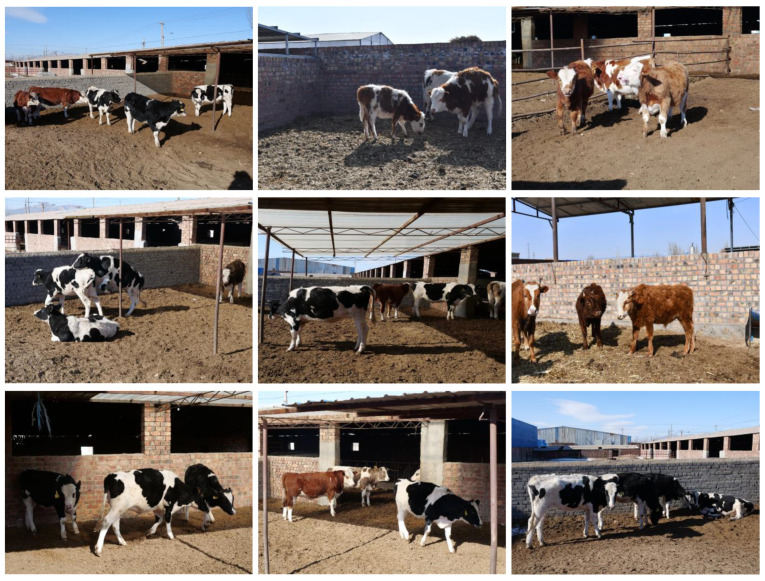
Real farming scenarios in the CAIDRE dataset. There are many identities such as standing, lying down, or walking. Some of them are partially obstructed by the farming structure or other animals. The breed of cattle is not limited to Simmental, but also includes Holstein Friesian cattle. Best viewed in color.

To facilitate the engineering application, we created the CAIDRE dataset, as a validation supplement to validate the performance of the model trained on the CNSID100 dataset under a realistic environment, such as the presence of mutual occlusion and more complicated background conditions. This was taken from 382 images including 27 identities, from fixed cameras in several real farm scenarios, as shown in Figure 7. The breed was not only limited to Chinese Simmental, but also included Holstein Friesian cattle.

**Figure 8:**
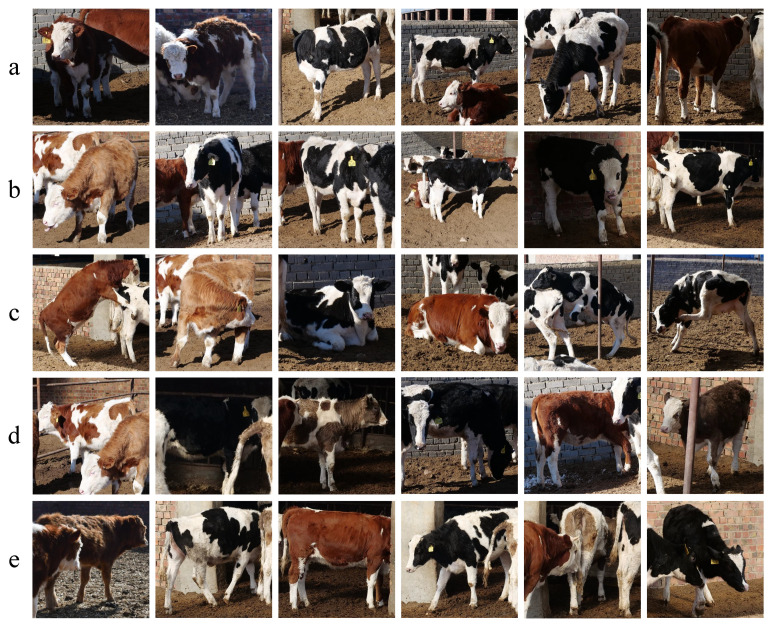
CAIDRE dataset examples. The CAIDRE dataset contains 27 individuals, including not only Chinese Simmental, but also Holstein Friesian cattle in several scenarios. It faces the difficulties of identification for a more complex background (Lines **a** and **b**), different postures (Line **c**), and partially occlusion with structures or other animals (Lines **d** and **e**). The samples are more complicated and realistic with real farming conditions than in the CNSID100 dataset. Best viewed in color.

Images in the CAIDRE dataset were split into a ratio of 3:1 for training and testing. The test set included 91 images, part of which is show in Figure 7. About 450 training samples for the *k*-NN classifier in the engineering application were detected and cropped from the training set in CAIDRE by YOLOv5s [30], including a more complex background (Lines a and b in Figure 8), different postures (Line c in Figure 8), partially obscuring each other (Lines d and e in Figure 8), and more complicated than CNSID100. Details are shown in Figure 8. Then, the training samples were input into ResNet50 to extract features and to create the features dataset for the *k*-NN classifier with k=2.

**Figure 9:**
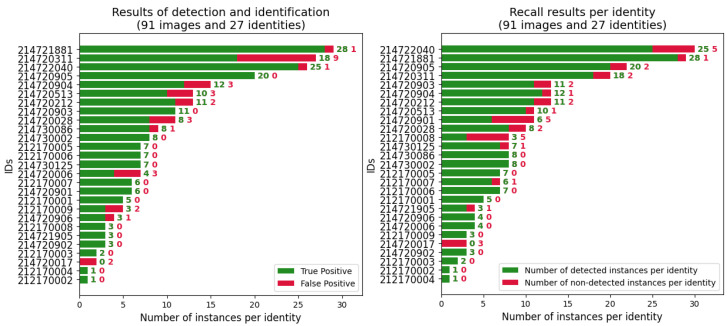
Precision and recall results. (**Left**) The precision of the detection and identification per identity with the mean precision achieving 88.14%. (**Right**) The recall of detection and identification per identity with a mean value of 86.43%. Best viewed in color.

Rather than retraining the model with CAIDRE, we directly extracted the features of the targets in the CAIDRE images using ResNet50 and weight training on half of the identities in CNSID100 with the best accuracy of 98.97% in Section 5.4. For cattle detection, YOLOv5s with the proposed weights achieved a 99.1% mAP. Based on the performance of the object detection with YOLOv5s, the precision of our pipeline for detection and identification achieved 88.14%, and the recall was 86.43% at a 0.5 intersection over union, as shown in Figure 9. The time spent on object detection was 8.9 ms per image, and that spent on identification, including the processing of feature extraction and *k*-NN classification, was 21.1 ms per target. The details are shown in Table 5.

**Figure 10:**
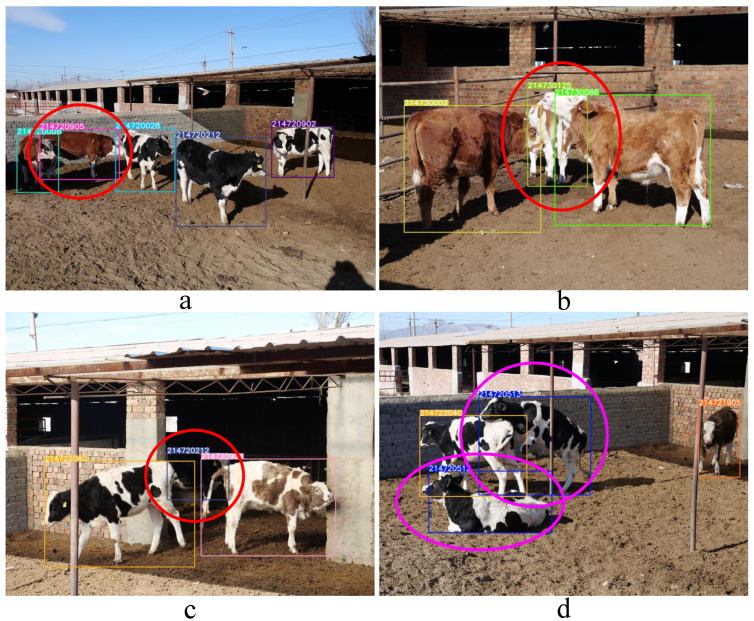
Engineering application results. It is shown that our proposed approach has the ability to generalize to new breeds and more realistic and complicated scenarios. It correctly identified instances obstructed by farming structures or other animals (the targets circled in red in (**a**–**c**)). It also correctly recognized the breeds that had diverse postures (the targets circled in pink in (**d**)). Best viewed in color.

Although our proposed loss supervised the model with images in the CNSID100 dataset with standing posture and no occlusion with farm structures or other individuals, it was still effective at extending to identify new individuals in more complex conditions, even with a variety of postures and obstructed instances. The typical results of the detection and identification are shown in Figure 10.

**Figure 11:**
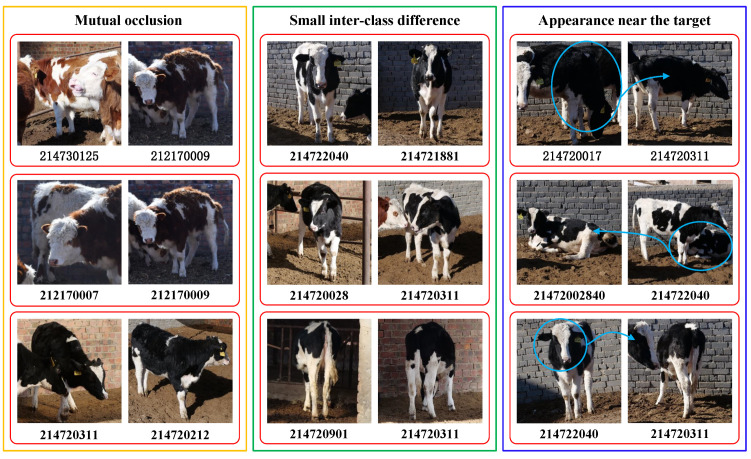
Typical identification errors in CAIDRE with ResNet50. The left image of the pairs in the red box is the test identity, and the right one is its wrongly predicted result. Mutual occlusion, high similarities, and appearance around the target are the main causes of misidentification. Best viewed in color.

However, some of the typical identification errors in the application experiments are listed in Figure 11. As is shown in Figure 11, there were mainly three types of errors caused by mutual occlusion, high similarities, and appearance around the target, all of which are common in a realistic farming environment. In addition, the mean value of precision and recall was also affected to some extent due to cattle detection errors.

## 7. Discussion

In this paper, we introduced SoftMax with multiple centers to learn agent points for each individual to capture more local clusters and constructed agent triplet loss with an anchor point and its positive and *K*-nearest negative agents to learn embeddings without an extra mining strategy. By joint supervision with our proposed multi-center agent loss, more discriminative features were learned to obtain SOTA solutions in cattle identification tasks under the open-set protocol. We created the CNSID100 dataset with multi-viewpoint images of cattle in a nearly natural state and will make it available publicly for further applications for cattle identification/re-identification and verification tasks. Extensive experiments, comparing triplet loss, ArcFace, and Softtriple loss on our CNSID100 dataset and with SoftMax-based reciprocal triplet loss on the OpenCow2020 dataset, convincingly demonstrated the effectiveness of the proposed approach. Taking advantage of the coat pattern as a biometric identifier to perform automated visual identification of cattle with an image from any viewpoint is very helpful for continuous monitoring of cattle in a natural farming state. Moreover, the open-set protocol is able to pave the way for the model to generalize to new farms and new breeds without any retraining, which is of vital importance for actual application tasks in real livestock farming.

An engineering application pipeline was given in this paper to perform detection and identification tasks in real farming. However, high similarity between identities of the same view, intra-class variety with the change of views and postures, and mutual occlusions have been the main difficulties for identification in the real farming environment. Thus, we will look towards tracking with multiple cameras to build seamless video-based pipelines for detection and identification in a real environment. The use of multiple cameras, complementing each other, would be helpful for identifying and tracing from a better viewpoint with less or no occlusion.

How robust this approach will be remains to be evaluated for the identification of new breeds with a greater variety of views, postures, backgrounds, and mutual occlusions in realistic farming conditions. Increasing the number of individuals via continuous data sampling is helpful to reinforce the scalability of the model to new herds on new farms. As the number of images and individuals in the real farming environment increases, target annotation will be the most time-consuming task. Thus, the approach of weakly supervised learning will be the main focus of our current and future research.

Based on continuous cattle detection and identification, we will conduct research on behavior detection and recognition for welfare and health assessment. With the standard deployment of cattle detection, identification, and behavior recognition, it is possible to utilize monitoring on farms within company or government networks to provide the services of production monitoring, early disease detection, and animal science research for precision livestock farming.

## Figures and Tables

**Table 1:** Datasets for visual cattle identification.

Author	Year	Identities	Images/Videos	Details
Allen et al. [9]	2008	869	1738	Retina
Lu et al. [8]	2014	6	60	Iris
Santosh Kumar et al. [4]	2017	500	5000	Muzzle
Wenyong Li [14]	2017	22	1965	Tailhead images
William Andrew et al. [13]	2021	46	4376	Dorsal images
Our dataset	2021	100	11,635	Multi-view images

**Table 2:** Ablation study of multi-center agent loss on CNSID100 with 50% individuals unseen.

	Average Accuracy (%):[Minimum, Maximum]
SoftMax with single center	96.84:[96.5, 97.17]
Single center agent loss	96.96:[96.44, 97.47]
SoftMax with multiple centers	97.29:[96.86, 97.71]
Multi-center agent loss	98.55:[98.13, 98.97]

**Table 3:** Results on CNSID100 with 50% individuals unseen.

	Average Accuracy (%):[Minimum, Maximum]
Triplet loss [18]	93.45:[91.72, 95.17]
ArcFace [24]	97.59:[96.74, 98.43]
Softtriple [26]	97.59:[97.22, 97.95]
Ours	98.55:[98.13, 98.97]

**Table 4:** Result on OpenCow2020 with 50% individuals unseen.

	Average Accuracy (%):[Minimum, Maximum]
SoftMax-based reciprocal triplet loss [13]	98.19:[97.58, 98.79]
Ours	98.59 [97.99, 99.19]

**Table 5:** Implementation details for the engineering application.

Results of implementation		
mAP@0.5	99.1%	Cattle detection
Precision	88.14%	Detection and identification
Recall	86.43%	Detection and identification
Detection time	8.9 ms per image	
Recognition time	21.1 ms per target	Feature extraction and *k*-NN classification
Hardware Configuration		
CPU: Intel i9	GPU: NVIDIA 2080TI	Memory: 64 G
Software Configuration		
Ubuntu 18.06	Python 3.6	Pytorch 1.7.1

## Data Availability

The datasets used and analyzed in the current study will be available from the corresponding author after the paper is accepted.

## Abbreviations

The following abbreviations are used in this manuscript:

DCNNs	Deep Convolutional Neural Networks
DML	Deep Metric Learning
FR	Face Recognition
SOTA	State-Of-The-Art
*K*-NNAT loss	*K*-Nearest Negative Agent Triplet loss
